# Survival, Failure Modes, and Risk Factors After Limb Salvage Surgery with Modular Megaprosthetic Reconstruction

**DOI:** 10.3390/cancers18203278

**Published:** 2026-10-12

**Authors:** Panayiotis Gavriil, Vasileios Karampikas, Stavros Goumenos, Anastasios Roustemis, Ioannis Trikoupis, Philippos Orfanos, Pagona Lagiou, Sotirios Tsiodras, Vasileios Kontogeorgakos, Panayiotis Papagelopoulos

**Affiliations:** 1 First Department of Orthopaedics, ATTIKON University General Hospital, National and Kapodistrian University of Athens, 12462 Athens, Greece; gavriilpan@med.uoa.gr (P.G.); vas.karampikas@gmail.com (V.K.); stgoumenos@gmail.com (S.G.); vkonto@med.uoa.gr (V.K.); 2 Leeds Major Trauma Centre, Leeds Teaching Hospitals NHS Trust, Leeds LS1 3EX, UK; 3 Department of Hygiene, Epidemiology and Medical Statistics, School of Medicine, National and Kapodistrian University of Athens, 75 Mikras Asias Street, 11527 Athens, Greece; 4 Department of Epidemiology, Harvard T.H. Chan School of Public Health, 677 Huntington Avenue, Boston, MA 02115, USA; 5 Fourth Department of Internal Medicine, ATTIKON University General Hospital, School of Medicine, National and Kapodistrian University of Athens, 12462 Athens, Greece

**Keywords:** sarcoma, limb salvage, megaprosthesis, complications, complex joint reconstruction, periprosthetic infection, risk factors

## Abstract

Background/Objectives: Modular megaprosthetic reconstruction is a central limb-salvage strategy after major skeletal resection, but long-term outcomes remain limited. This study aimed to evaluate patient and implant survival, complication burden, Henderson failure modes, and associated risk factors. Methods: We retrospectively analyzed 340 consecutive patients undergoing modular megaprosthetic reconstruction at a tertiary referral center from 2003 to 2024 for primary malignant bone tumors, metastatic disease, or complex revision- or trauma-related indications. Kaplan–Meier methods estimated patient, implant, and complication-free survival. Implant failure and first recorded Henderson failure modes were additionally assessed using competing-risk methods. Cox regression evaluated risk factors, with multivariable modelling focused on Type IV infection. Results: Median follow-up was 5 years. Patient survival was 71.4% at 5 years and 61.3% at 10 years and was poorer in metastatic disease (*p* < 0.001). Implant survival was 78.0% and 70.3%, respectively. Complications occurred in 133 patients (39.1%). Type IV infection was the most frequent first failure mode (49, 14.4%), followed by Type I soft-tissue failure (40, 11.8%). The cumulative incidence of infection was 14.6% at 5 years and 15.3% at 10 years. Previous surgeries at the operative site (hazard ratio [HR] 2.30 per additional surgery, 95% confidence interval [CI] 1.90–2.80), operative time (HR 1.005/min, 95% CI 1.002–1.008), and diabetes mellitus (HR 2.30, 95% CI 1.25–4.25) were independently associated with infection and remained significant after expanded adjustment. Conclusions: Implant survival remained 70.3% at 10 years, but complications were frequent. Infection was the dominant first failure mode, and prior local surgery, longer operative time, and diabetes identified higher-risk patients. These findings support individualized risk assessment, preventive planning, and long-term surveillance.

## Simple Summary

Megaprostheses play a crucial role in the reconstruction of major bone and joint defects after tumor resection. They represent a reliable surgical solution with favorable functional outcomes. However, reported complication rates remain high. We reviewed outcomes in 340 patients treated at a specialist referral center for bone tumors and complex joint reconstruction. We examined patient survival, implant durability, and the types and timing of complications. Approximately four in ten patients experienced a complication, with infection and soft-tissue-related problems the most common first complications. Previous operations at the same site, longer surgery, and diabetes were associated with infection. These findings highlight the importance of considering patient prognosis alongside implant durability and planning long-term follow-up. They also provide information to support discussions with patients about the expected course and risks of reconstruction.

## 1. Introduction

Advances in oncologic therapy, surgical technique, and implant design have made limb-salvage surgery the preferred approach for malignant bone tumors when wide resection can be achieved without compromising survival [1,2,3,4]. Modular megaprostheses provide immediate stability and intraoperative flexibility for reconstructing large bone and joint defects, thereby facilitating early mobilization [5,6,7,8]. Although their principal indication remains reconstruction after primary malignant bone-tumor resection, their use now extends to metastatic disease, locally aggressive benign tumors, and selected complex revision- or trauma-related defects [5,9]. Long-term series support durable limb salvage and clinically meaningful function despite the complexity of these procedures [3,10].

However, failure after megaprosthetic reconstruction remains common. The Henderson classification distinguishes Type I soft-tissue failure, Type II aseptic loosening, Type III structural failure, Type IV infection, and Type V tumor progression and emphasizes that failure patterns vary by anatomical site and over time [11,12]. In a systematic review of 2721 patients, Thornley et al. reported reoperation in 47%; mechanical complications predominated, infection generally occurred earlier, and aseptic loosening accumulated later [13]. Institutional series have similarly reported overall failure rates of approximately 29% [14,15]. Complications may also recur: after revision for a first failure, Theil et al. observed a second complication in 49% of patients at a median of 17 months [16]. Megaprosthetic outcomes should therefore be assessed beyond implant survival alone, with attention to failure type, timing, and clinical context.

Such evidence is difficult to generate because primary malignant bone tumors are rare and complex reconstructions are concentrated in specialized referral centers, where treatment volume and multidisciplinary expertise have been associated with improved oncologic outcomes [7,17,18,19,20]. In this setting, randomized studies are difficult to conduct, and large longitudinal institutional cohorts remain an important source of evidence [11,13]. Existing studies are often small or narrowly focused on one anatomical site, diagnosis, implant system, or endpoint and use inconsistent definitions and follow-up reporting [13]. In addition, outcomes reflect interrelated patient-, oncologic-, anatomical-, surgical-, and implant-related factors that may be incompletely understood when examined separately [3,21,22]. A consecutive referral-center cohort encompassing oncologic and selected revision- or trauma-related indications can therefore provide real-world evidence on modular megaprosthetic reconstruction across different clinical settings. Evaluating patient-, surgical-, and implant-related factors together, while reporting survival by indication, provides clinical context for interpreting both patient and reconstruction outcomes.

Accordingly, this study aimed to: (1) estimate patient survival, implant survival, and complication-free survival and examine differences by surgical indication, (2) describe Henderson failure frequencies overall and by anatomical site and characterize their timing using overall competing-risk analyses and (3) identify factors associated with postoperative complications.

## 2. Patients & Methods

### 2.1. Study Design and Setting

This retrospective cohort study included consecutive patients who underwent modular megaprosthetic reconstruction at a tertiary academic referral center for orthopedic oncology and complex limb reconstruction between 2003 and 2024. Data were obtained from a prospectively maintained institutional database and analyzed retrospectively to evaluate patient and implant survival, postoperative complications, Henderson failure modes, and associated risk factors. The proximal femoral subgroup includes patients previously reported in our institutional series of proximal femoral megaprosthetic reconstruction [23]. Reporting followed the STROBE recommendations for observational cohort studies.

### 2.2. Study Population

Consecutive patients were eligible for inclusion if they underwent limb-salvage reconstruction with a modular megaprosthesis for a major skeletal defect involving the proximal humerus, proximal femur, distal femur, proximal tibia, or combined distal femur/proximal tibia reconstruction. No age restriction was applied, and modular expandable prostheses were eligible when they met the remaining study criteria. Surgical indications included primary malignant bone tumors, metastatic bone disease, and complex revision- or trauma-related reconstructions.

Patients with death, implant failure, or a postoperative complication within 12 months were retained, whereas event-free patients required at least 12 months of postoperative follow-up. Pelvic endoprostheses, non-modular custom reconstructions, anatomical sites outside the predefined locations, and cases with insufficient clinical documentation were excluded.

### 2.3. Data Collection and Variables

Follow-up was calculated from index megaprosthetic reconstruction to the last documented clinical contact or death. Recorded variables included demographic and clinical factors: age, sex, body mass index, smoking status, diabetes mellitus, Charlson Comorbidity Index, and ASA score where available; oncologic and treatment-related factors: surgical indication, tumor diagnosis, chemotherapy, radiotherapy, and margin status; and surgical or implant-related factors: anatomical site, prosthesis system, cemented fixation, silver coating, Trevira attachment tube (implantcast GmbH, Buxtehude, Germany) use, prosthesis length, specimen dimensions, operative time, hospital stay, transfusions, and previous surgeries at the operative site before index reconstruction. Estimated intraoperative blood loss and a standardized classification of immune status at index reconstruction were not consistently available for analysis. Transfusion requirements were recorded separately and were not treated as a direct measure of intraoperative blood loss.

### 2.4. Outcome Definitions and Complication Classification

The primary time-to-event outcomes were patient survival, implant survival, and time to first postoperative complication. Patient survival was measured from index reconstruction to death from any cause or last follow-up. Implant failure was defined as revision requiring exchange or removal of the prosthesis, with implant survival measured from index reconstruction to implant failure or last follow-up.

Complications were classified as Henderson Type I soft-tissue failure, Type II aseptic loosening, Type III structural failure, Type IV infection, or Type V tumor progression. For patients with temporally related or causally linked complications, one primary Henderson failure mode was assigned to avoid double-counting.

The Henderson failure-mode analyses used a first-event framework. Subsequent or recurrent complications were not included in the failure-mode frequency or cause-specific cumulative-incidence analyses. The primary recorded Henderson failure mode and subtype-specific risk-factor analyses were considered secondary and exploratory outcomes.

### 2.5. Statistical Analysis

Descriptive statistics were used to summarize baseline, oncologic, surgical, and postoperative characteristics. Continuous variables were presented as mean ± standard deviation or median with interquartile range, as appropriate, and categorical variables as counts and percentages. Baseline characteristics were summarized for the overall cohort and by grouped anatomical site. No formal hypothesis testing was performed for baseline differences in Table 1. Outcomes were additionally summarized descriptively for each of the five anatomical reconstruction sites (Table S1). Overweight (25 ≤ BMI < 30 kg/m^2^) and obesity (BMI ≥ 30 kg/m^2^) were additionally described among adults (≥18 years) with any postoperative complication or first primary Type IV infection. Selected patient and perioperative characteristics and crude complication proportions were additionally summarized by sex using available observations; no formal between-sex hypothesis tests were performed.

Kaplan–Meier methods estimated patient survival, implant survival, and complication-free survival at 1, 3, 5, and 10 years. Predefined groups, including surgical indication and prosthesis type where applicable, were compared using log-rank tests. Implant failure was also evaluated using cumulative incidence functions with death as a competing event. For each first primary Henderson failure mode, death and the remaining failure types were treated as competing events.

Univariable Cox proportional hazards models evaluated predictors of time to first postoperative complication. Type I and Type II analyses were restricted to clinically relevant anatomical subgroups, and Type III events were insufficient for reliable risk-factor modelling. Univariable and subtype-specific analyses were exploratory, without adjustment for multiple comparisons, and were interpreted using effect estimates, 95% confidence intervals, event counts, and clinical plausibility. Complete outputs are provided in Supplementary Table S2.

Because Type IV infection was the most frequent individual failure mode and provided the largest number of subtype-specific events, it was selected for multivariable Cox regression. Candidate variables were considered on the basis of clinical relevance, temporal appropriateness, univariable findings, and the number of available events. Nested models were examined, and the primary model was intentionally restricted to three clinically coherent predictors—previous surgeries at the operative site, operative time, and diabetes mellitus—to reduce the risk of overfitting. An expanded clinically adjusted sensitivity model additionally included grouped anatomical site, silver coating, and cemented fixation. Both multivariable Type IV infection models included 337 patients with complete covariate data and 49 infection events. For Type IV cause-specific Cox modelling, patients with a different first primary recorded Henderson failure mode were censored at the time of that competing failure. Hazard ratios were reported with 95% confidence intervals. Previous surgeries at the operative site were modelled per additional previous operation, and operative time was modelled per minute. Sensitivity analyses used a categorical previous-operation count (0, 1, or ≥2) and assessed nonlinearity by adding a squared count term to the original model, evaluated using a likelihood-ratio test.

The proportional hazards assumption was assessed using Schoenfeld residuals and graphical methods. Complete-case analysis was used for regression models, and no statistical imputation was performed. All tests were two-sided, and *p* values < 0.05 were considered statistically significant. Analyses were performed using Stata 13 (StataCorp LLC, College Station, TX, USA).

### 2.6. Ethics Approval

The Scientific Council and Ethics Committee of ATTIKON University Hospital approved the study (639/11.09.2023) and waived individual informed consent because anonymized, routinely collected data were analyzed retrospectively. Data were handled in accordance with institutional data-protection procedures and applicable privacy regulations.

### 2.7. Data Availability Statement

The data supporting the findings of this study are available from the corresponding author upon reasonable request, subject to institutional, ethical, and privacy restrictions.

## 3. Results

### 3.1. Cohort Characteristics

A total of 340 patients undergoing modular megaprosthetic reconstruction were included. Patient selection is summarized in Figure S1. The mean age was 55.2 ± 19.7 years, and 186 patients (54.7%) were male. Surgical indications included primary malignant bone tumors in 175 patients (51.5%), metastatic disease in 99 patients (29.1%), and revision or trauma-related reconstruction in 66 patients (19.4%).

The most common anatomical site was the proximal femur. Overall, MUTARS prostheses (Modular Universal Tumor and Revision System; implantcast GmbH, Buxtehude, Germany) were used in 213 cases (62.6%) and Stanmore prostheses (Modular Endoprosthetic Tumour System; Stanmore Implants Worldwide Ltd., Elstree, UK; now Stryker) in 127 cases (37.4%). Silver-coated implants were used in 155 patients (45.6%), and cemented fixation was performed in 211 cases (62.1%). Median operative time was 150 min [IQR 130–180], and median follow-up duration was 5 years [IQR 3–9; range 0.1–20]. Previous operations at the operative site had a median of 0 [IQR 0–1; range 0–5]. There were 217 patients (63.8%) with no previous operation, 84 (24.7%) with one, 32 (9.4%) with two, and 7 (2.1%) with three or more. Baseline, oncologic, and surgical characteristics overall and by grouped anatomical site are summarized in Table 1.

### 3.2. Patient Survival and Implant Survival

**Figure 1:**
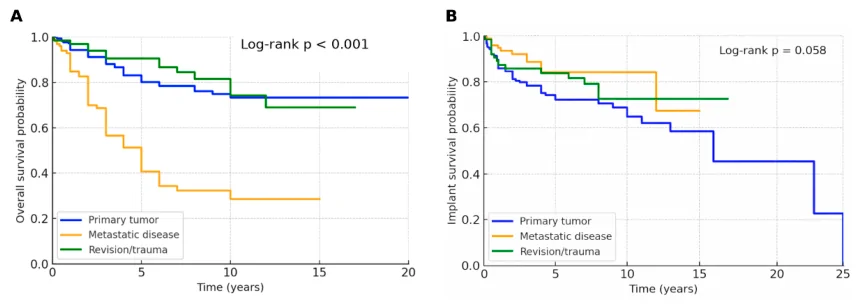
Kaplan–Meier estimates of patient and implant survival according to surgical indication. (**A**) Overall patient survival stratified by surgical indication, showing significantly poorer survival among patients treated for metastatic disease (log-rank *p* < 0.001). (**B**) Implant survival stratified by surgical indication (log-rank *p* = 0.058).

By final follow-up, 106 patients (31.2%) had died. Kaplan–Meier estimated overall patient survival was 92.0% at 1 year, 79.7% at 3 years, 71.4% at 5 years, and 61.3% at 10 years. Patient survival differed significantly by surgical indication (log-rank *p* < 0.001), with the poorest survival observed in patients treated for metastatic disease. Five- and ten-year patient survival were 80.1% and 73.3% for primary tumor cases, 40.7% and 28.7% for metastatic disease, and 90.6% and 74.2% for revision/trauma cases, respectively (Table 2; Figure 1A).

A total of 78 implant failures (22.9%) occurred during follow-up. Kaplan–Meier estimated overall implant survival was 88.7% at 1 year, 82.8% at 3 years, 78.0% at 5 years, and 70.3% at 10 years. Implant survival did not differ significantly by surgical indication (log-rank *p* = 0.058).

Five- and ten-year implant survival were 72.2% and 64.7% for primary tumor cases, 84.2% and 84.2% for metastatic disease, and 83.8% and 72.7% for revision/trauma cases, respectively (Table 2; Figure 1B). The apparently favorable implant survival in metastatic disease should be interpreted in the context of competing mortality, which was further addressed using competing-risk analysis.

### 3.3. Competing-Risk Analysis of Implant Failure

**Figure 2:**
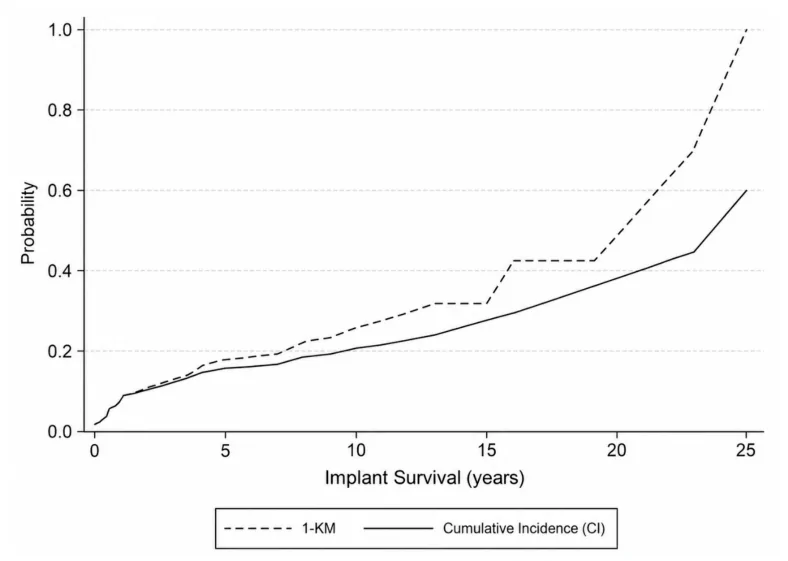
Competing-risk analysis of implant failure. Kaplan–Meier-derived implant failure estimates (1 − KM) are compared with cumulative incidence estimates accounting for death as a competing event. The cumulative incidence function provides lower estimates of implant failure probability, consistent with the effect of patient mortality as a competing event.

Because death precludes the subsequent observation of implant failure, implant failure probability was additionally evaluated using a competing-risk framework with death treated as a competing event. Figure 2 compares the Kaplan–Meier-derived estimate of implant failure probability (1 − KM) with the cumulative incidence function. The cumulative incidence estimates were lower than the corresponding 1 − KM estimates, supporting the interpretation that conventional Kaplan–Meier methods may overestimate implant failure probability when patient mortality is not accounted for as a competing event.

This consideration is particularly relevant in a heterogeneous cohort including patients with metastatic disease, in whom mortality was substantially higher than in the other indication groups. Therefore, implant-survival estimates should be interpreted alongside patient survival and competing-risk estimates rather than as isolated mechanical survival outcomes.

### 3.4. Overall Complication Burden

During follow-up, 133 of 340 patients (39.1%) experienced at least one postoperative complication. Among patients who developed a complication, the observed median time to first complication was 1.0 year [IQR 0.4–2.1].

Kaplan–Meier estimates of complication-free survival were 76.7%, 66.5%, 62.3%, and 56.3% at 1, 3, 5, and 10 years, respectively. Complication-free survival did not differ significantly by surgical indication (log-rank *p* = 0.930) or by prosthesis type (log-rank *p* = 0.117). The corresponding Kaplan–Meier curves are presented in Figure S2. Thus, postoperative complications represented a substantial long-term burden after megaprosthetic reconstruction, but this burden was not clearly explained by surgical indication or prosthesis system alone.

### 3.5. Henderson Failure Modes

Among the 133 patients who experienced at least one postoperative complication, each patient was classified according to the primary recorded Henderson failure mode to avoid double-counting of temporally related or causally linked complications. Type IV infection was the most frequent failure mode, occurring in 49 patients (14.4% of the total cohort; 36.8% of patients with complications). Type I soft-tissue failure was the second most common category, occurring in 40 patients (11.8% of the total cohort; 30.1% of patients with complications).

Less frequent failure modes included Type II aseptic loosening in 17 patients (5.0% of the total cohort; 12.8% of patients with complications), Type V tumor progression in 17 patients (5.0%; 12.8%), and Type III structural failure in 10 patients (2.9%; 7.5%). Thus, infection and soft-tissue failure together accounted for approximately two-thirds of primary recorded complications. The distribution of Henderson failure modes overall and by grouped anatomical site is shown in Table 3. Descriptive outcomes by individual anatomical reconstruction site are provided in Supplementary Table S1.

### 3.6. Cumulative Incidence of Henderson Failure Modes

**Figure 3:**
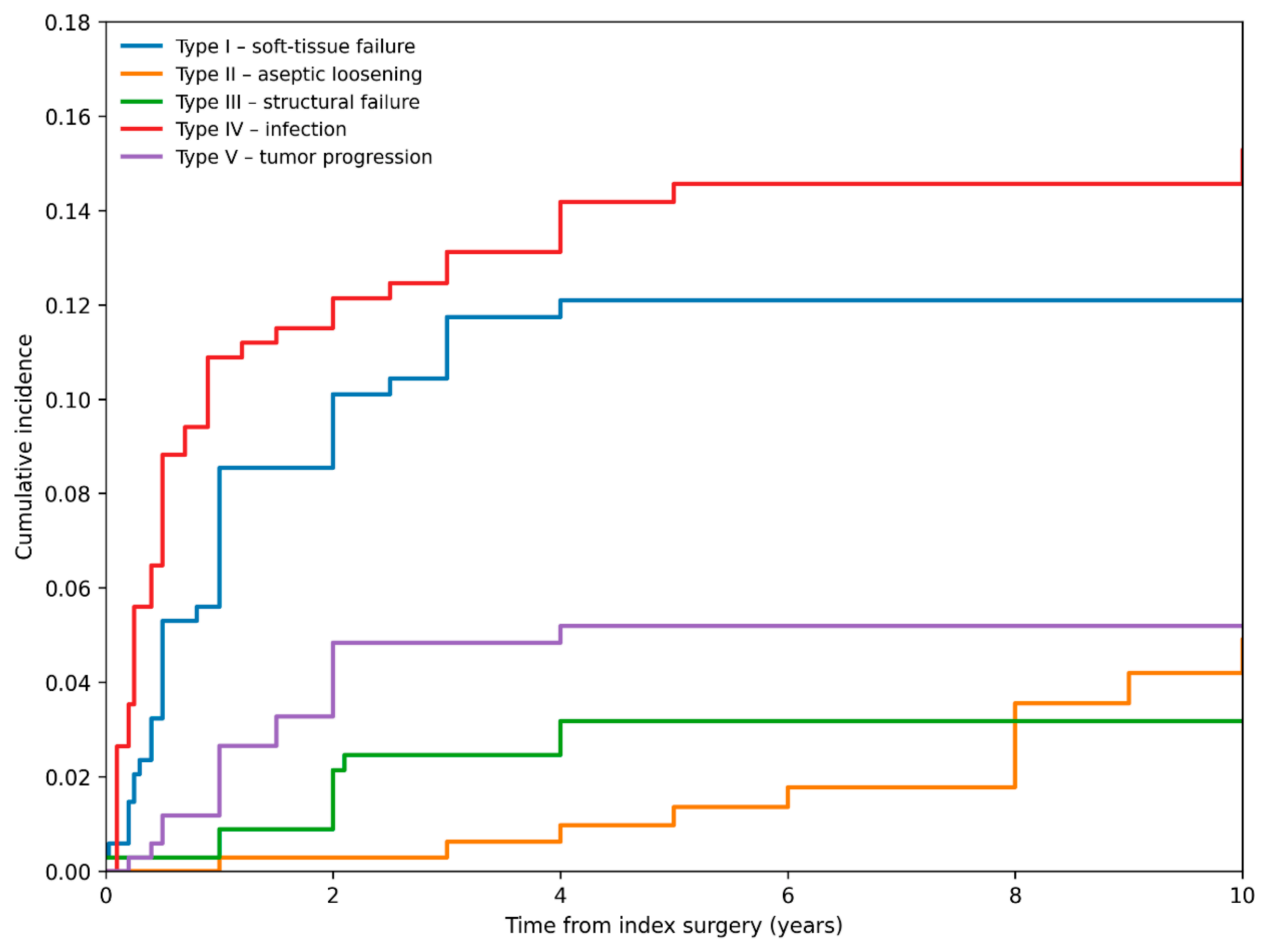
Cumulative incidence of first primary recorded Henderson failure modes. Competing-risk analysis showing the cumulative incidence of Type I soft-tissue failure, Type II aseptic loosening, Type III structural failure, Type IV infection, and Type V tumor progression, with death and the remaining Henderson failure types treated as competing events.

In competing-risk analysis based on the first primary recorded Henderson failure mode, Type IV infection showed the highest cumulative incidence over time, followed by Type I soft-tissue failure (Figure 3). The cumulative incidence of Type IV failure was 10.9% at 1 year, 13.1% at 3 years, 14.6% at 5 years, and 15.3% at 10 years. The corresponding cumulative incidence estimates for Type I failure were 8.5%, 11.7%, 12.1%, and 12.1%, respectively.

Other failure modes occurred less frequently. The cumulative incidence of Type II aseptic loosening was 0.3% at 1 year, increasing to 4.9% at 10 years, suggesting a later-occurring pattern. Type III structural failure reached 3.2% by 5 years and remained stable thereafter, while Type V tumor progression reached 5.2% by 5 years and did not further increase during later follow-up. Overall, this competing-risk analysis confirmed that infection represented the dominant first failure mode in the cohort, whereas aseptic loosening tended to accumulate later in follow-up.

### 3.7. Risk-Factor Analyses

#### 3.7.1. Univariable Risk-Factor Analyses

Univariable Cox proportional hazards analyses were performed to explore factors associated with postoperative complications. Complete univariable model outputs are provided in Table S2. For any postoperative complication, larger specimen dimensions (HR 1.003, 95% CI 1.001–1.006, *p* = 0.013), longer hospital stay (HR 1.04, 95% CI 1.02–1.05, *p* < 0.001), diabetes mellitus (HR 1.84, 95% CI 1.25–2.69, *p* = 0.002), and longer operative time (HR 1.004 per minute, 95% CI 1.002–1.007, *p* = 0.001) were associated with increased hazard of complication. Because hospital stay is a postoperative variable, this association was interpreted as reflecting postoperative course and overall case complexity rather than as a baseline causal predictor.

Subtype-specific analyses showed more limited findings. For Type I soft-tissue failure, the analysis was restricted to proximal humeral and proximal femoral reconstructions. Within this subgroup, anatomic proximal humeral designs were associated with a markedly higher hazard of soft-tissue failure compared with reverse-type designs (HR 7.1, 95% CI 2.0–25.4, *p* = 0.002). For Type II aseptic loosening, no examined variable reached statistical significance, although there was a non-significant trend toward lower hazard with Stanmore compared with MUTARS prostheses (HR 0.30, 95% CI 0.08–1.08, *p* = 0.065). Type III structural failure was too infrequent to support robust risk-factor modelling.

For Type IV infection, several variables were associated with increased hazard in univariable analysis, including longer operative time (HR 1.008 per minute, 95% CI 1.005–1.011, *p* < 0.001), a greater number of previous surgeries at the operative site (HR 2.63 per additional surgery, 95% CI 2.20–3.14, *p* < 0.001), diabetes mellitus (HR 3.54, 95% CI 2.01–6.22, *p* < 0.001), higher BMI (HR 1.07 per kg/m^2^, 95% CI 1.01–1.13, *p* = 0.036), higher ASA score (HR 1.39, 95% CI 1.09–1.79, *p* = 0.009), and transfusions (HR 1.43, 95% CI 1.26–1.63, *p* < 0.001). Age, prosthesis type, Trevira use, silver coating, cemented fixation, smoking, patient demands, and the analyzed implant-ratio variables were not significantly associated with infection. The Charlson Comorbidity Index showed a borderline association (HR 1.15, 95% CI 0.99–1.33, *p* = 0.076). Among adult patients with at least one complication, 58/122 (47.5%) were overweight and 40/122 (32.8%) had obesity. Among adults with Type IV infection as the first primary recorded failure, 18/46 (39.1%) were overweight and 20/46 (43.5%) had obesity. Descriptive summaries of BMI, diabetes prevalence, previous local operations, and operative time were broadly comparable between male and female patients. Any postoperative complication occurred in 72/186 males (38.7%) and 61/154 females (39.6%), while first-primary Type IV infection occurred in 24/186 (12.9%) and 25/154 (16.2%), respectively. These percentages represent crude observed proportions rather than adjusted sex-specific risks.

#### 3.7.2. Type IV Infection Multivariable Model

**Figure 4:**
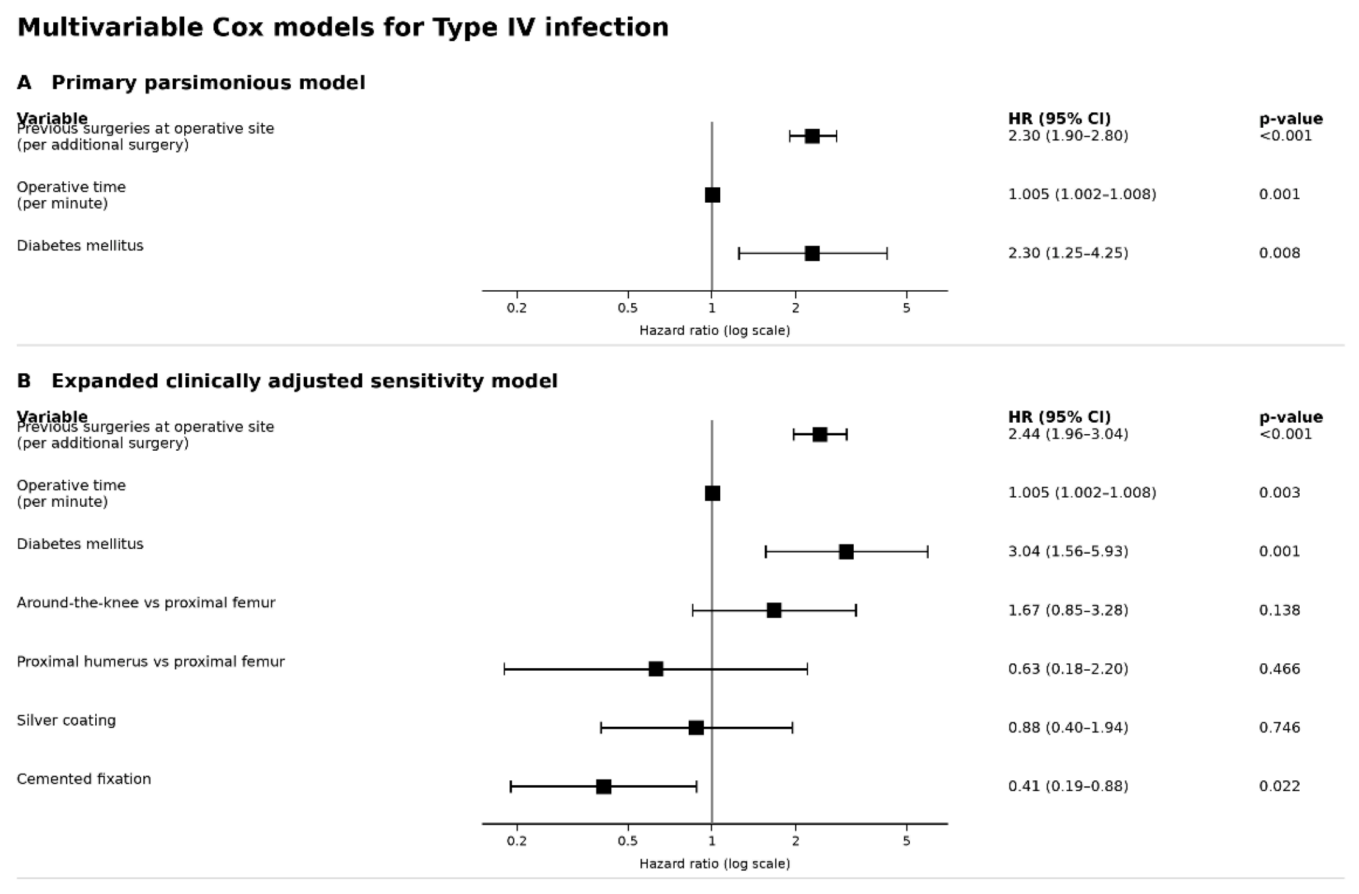
Multivariable Cox regression models for Type IV infection. (**A**) Primary parsimonious model including previous surgeries at the operative site, operative time, and diabetes mellitus. (**B**) Expanded clinically adjusted sensitivity model additionally including grouped anatomical site, silver coating, and cemented fixation. Hazard ratios are shown on a logarithmic scale with 95% confidence intervals. In Panel (**B**), proximal femur was the reference category for grouped anatomical site; around-the-knee reconstructions included distal femur, proximal tibia, and combined distal femur/proximal tibia reconstructions.

Because Type IV infection was the most frequent individual Henderson failure mode and provided the most stable number of events for adjusted modelling, this endpoint was selected for multivariable Cox regression. In the primary parsimonious model, the number of previous surgeries at the operative site, operative time, and diabetes mellitus remained independently associated with Type IV infection (Figure 4A). In the original log-linear specification, the estimated infection HR was 2.30 per additional previous operation (95% CI 1.90–2.80; *p* < 0.001). Longer operative time was also associated with infection (HR 1.005 per minute, 95% CI 1.002–1.008, *p* = 0.001), as was diabetes mellitus (HR 2.30, 95% CI 1.25–4.25, *p* = 0.008).

An expanded clinically adjusted sensitivity model was then fitted to assess the robustness of these associations after adjustment for grouped anatomical site, silver coating, and cemented fixation (Figure 4B). In this model, the main predictors remained significant: previous surgeries at the operative site (HR 2.44, 95% CI 1.96–3.04, *p* < 0.001), operative time (HR 1.005 per minute, 95% CI 1.002–1.008, *p* = 0.003), and diabetes mellitus (HR 3.04, 95% CI 1.56–5.93, *p* = 0.001). Compared with proximal femoral reconstruction, around-the-knee reconstruction showed a non-significant trend toward higher infection hazard (HR 1.67, 95% CI 0.85–3.28, *p* = 0.138), whereas proximal humeral reconstruction was not significantly associated with infection (HR 0.63, 95% CI 0.18–2.20, *p* = 0.466). Silver coating was not independently associated with infection (HR 0.88, 95% CI 0.40–1.94, *p* = 0.746). Cemented fixation showed an inverse adjusted association with Type IV infection (HR 0.41, 95% CI 0.19–0.88, *p* = 0.022), although this finding was considered exploratory given the non-randomized selection of fixation strategy. Overall, the sensitivity model supported the stability of the primary infection predictors after adjustment for clinically relevant anatomical and implant-related factors. Categorical sensitivity analysis supported higher infection hazards among patients with previous operations. In a separate sensitivity analysis, adding a squared previous-operation count term improved model fit (likelihood-ratio *p* < 0.001), indicating departure from a constant per-operation association.

## 4. Discussion

This study provides a comprehensive assessment of modular megaprosthetic reconstruction in a consecutive, real-world cohort of 340 patients treated at a tertiary orthopedic oncology referral center. Patient survival differed markedly by surgical indication, whereas implant durability remained acceptable but had to be interpreted alongside competing mortality. Complications accumulated over time, with infection and soft-tissue failure predominating earlier and aseptic loosening increasing later. Previous surgeries at the operative site, longer operative time, and diabetes mellitus were the most consistent independent predictors of Type IV infection. These findings support evaluating patient prognosis, reconstruction durability, failure mode, and timing together rather than implant retention alone.

Patient survival was poorest in patients with metastatic disease, consistent with previous cohorts [8,21,24,25]. This distinction is clinically important: long-term survivors require durable reconstruction over decades, whereas in metastatic disease the principal goal is a stable construct that permits early mobilization and is likely to remain functional for the patient’s remaining lifetime. Endoprostheses frequently outlive patients with metastatic or high-grade disease [26,27,28].

Overall implant survival was 78.0% at 5 years and 70.3% at 10 years, within the range reported in contemporary series. Comparisons depend strongly on the selected endpoint: Capanna et al. reported freedom from any further surgery of 75.9% and 66.2% at 5 and 10 years, respectively; Pala et al. reported survival free from any Henderson failure of 70% at 4 years and 58% at 8 years; and Jeys et al. reported 10-year survival of 75% for mechanical failure but 58% for revision from any cause [3,14,15]. Similar midterm durability has been reported in non-neoplastic salvage reconstruction, whereas older implants with longer follow-up have shown lower survival [2,29]. Sewell et al. further illustrated the effect of endpoint definition, reporting 100% 5-year survival with prosthesis removal as the endpoint but 56% when any subsequent operation was counted [30,31].

The apparently favorable implant survival in the metastatic group should not be interpreted as superior mechanical performance. Death removes patients from the risk of a later revision, and conventional Kaplan–Meier analysis may therefore overestimate the real-world probability of failure. Competing-risk studies support interpreting implant survival together with patient survival, particularly in metastatic disease [8,21,24,32,33,34].

Postoperative complications remained common, with complication-free survival declining to 62.3% at 5 years and 56.3% at 10 years, broadly consistent with previous reports [13,14,15,35,36,37]. Direct comparison remains difficult because studies use different endpoints, including any complication, reoperation, implant revision, or major Henderson failure. Complication-free survival did not differ significantly by indication or prosthesis system in our cohort, but this should not be interpreted as equivalence. Published comparisons are similarly inconsistent and likely reflect differences in patient survival, anatomical site, previous surgery, soft-tissue conditions, and reconstructive complexity rather than indication alone [15,29,38]. Current evidence is also insufficient to establish the superiority of one modular implant system [11].

Type IV infection and Type I soft-tissue failure were the leading first failure modes and together accounted for approximately two-thirds of complications, in keeping with contemporary series [11,14,15]. Our proportion of patients with first-primary Type IV infection (14.4%) falls within the deep infection frequencies of 5.1–23.6% summarized in a recent scoping review [39]. Direct comparison is limited by differences in case mix, follow-up, and outcome definitions, particularly our restriction to first-primary failures. In our cohort, Type I failure was more frequent after proximal humeral and femoral reconstruction than around the knee (Table 3). After proximal humeral reconstruction, shoulder instability is influenced by residual rotator cuff and deltoid function [40]. Proximal femoral series also identify instability or dislocation as an important complication [38,41]. Kurapatti et al. reported pooled dislocation/instability rates of 9% in nononcologic and 5% in oncologic proximal femoral replacements, with substantial heterogeneity [42]. The around-the-knee group showed the highest crude frequencies of infection, aseptic loosening, and structural failure. Individual-site descriptive outcomes are provided in Table S1, while the small proximal tibial and combined-reconstruction groups limit interpretation of site differences. Site-specific studies report a particularly high failure burden after proximal tibial reconstruction, related partly to limited soft-tissue coverage and extensor-mechanism reconstruction [43,44].

The frequency of soft-tissue failure highlights the importance of orthoplastic planning that integrates oncologic margins, skeletal reconstruction, soft-tissue coverage, and restoration of function [45]. Samà et al. emphasized upfront collaboration between oncologic and reconstructive surgeons to permit adequate sarcoma resection without restricting margins to facilitate closure [46]. Tension-free closure, dead-space management, and well-vascularized coverage aim to support healing and reduce wound breakdown and associated infection risk [45,46]. After proximal tibial replacement, a pedicled medial gastrocnemius flap can provide implant coverage and augment extensor-mechanism reconstruction [43]. The comparative effects of individual orthoplastic techniques were not evaluated in this cohort.

Failure timing also differed. Infection and soft-tissue failure accumulated mainly during earlier follow-up, whereas aseptic loosening increased more gradually, consistent with previous reports [13,47]. Infection nevertheless remains relevant in the long term, and late deep infection and reinfection support continued surveillance [37,48,49]. Finally, the first-event framework did not capture recurrent complications; previous studies found a subsequent complication in approximately half of patients after treatment of an initial failure, often within 1–2 years [36].

Risk-factor analyses suggested that postoperative complications were related mainly to host status and surgical complexity. Larger specimen dimensions and longer operative time were associated with any complication, likely reflecting more extensive resection, soft-tissue disruption, and technically demanding reconstruction rather than isolated causal effects. Similar associations between operative duration or reconstruction extent and early reoperation have been reported in PARITY secondary analyses and other megaprosthesis cohorts [21,50,51,52]. Diabetes was also associated with overall complications. Longer hospital stay was likewise associated with any complication in our univariable analysis. A PARITY secondary analysis reported an adjusted association between hospital length of stay and surgical-site infection (HR 1.03 per day, 95% CI 1.01–1.05) [51]. This association may reflect case complexity, early postoperative morbidity, or exposure to hospital-related hazards; complications may also prolong admission. Hospital stay therefore remains clinically relevant when interpreting postoperative recovery, but is not a baseline characteristic and cannot be assumed to precede all complications. These findings do not establish that shortening hospitalization alone would reduce complication risk. In the restricted proximal humeral analysis, anatomic designs were associated with a higher risk of Type I failure than reverse designs. This finding remains exploratory but is consistent with the greater dependence of anatomic reconstructions on the remaining rotator cuff and capsular structures [53]. No robust predictors were identified for aseptic loosening, and Type III events were too few for reliable modelling.

Type IV infection provided the most suitable endpoint for adjusted analysis. Previous operations may indicate scarred or poorly vascularized tissues, previous contamination, and increasing reconstructive complexity [28,54,55]. Operative time has also been associated with surgical-site infection and early reoperation, but probably reflects procedural magnitude at least in part [50,51,52]. Diabetes is an established infection risk factor in conventional arthroplasty but has been less consistently confirmed in megaprosthesis cohorts; its stable association in our analysis supports careful perioperative glycemic optimization [56,57]. Higher BMI and greater transfusion requirements were associated with infection in univariable analyses; these findings remain exploratory because neither variable was included in the adjusted models. Around-the-knee reconstruction showed only a nonsignificant trend, and no independent anatomical effect can therefore be concluded. Silver coating was not independently protective, consistent with the directionally favorable but statistically inconclusive meta-analysis by Gonzalez et al. [58]. Cemented fixation showed an inverse adjusted association, but this finding should remain hypothesis-generating because fixation was not randomized and current comparative evidence does not demonstrate a reliable independent effect on infection risk [59,60].

Strengths of this study include the consecutive 340-patient cohort, long-term follow-up, use of a prospectively maintained institutional database, standardized failure classification, and combined evaluation of patient survival, implant survival, competing failure modes, and complication risk factors.

Several limitations should be acknowledged. These are the retrospective, non-randomized design, the relatively limited minimum follow-up time, and the cohort’s heterogeneity. Although patients with documented early events were retained, requiring at least 12 months of follow-up for event-free patients may have introduced selection bias. Its effect on the reported estimates could not be reliably determined from the available records. Even though survival outcomes were reported separately by indication, pooled complication estimates and risk-factor associations describe the mixed reconstructive cohort rather than cancer-specific risks. Differences in indication, previous treatment, and patient prognosis may contribute residual confounding.

The long inclusion period may have introduced temporal heterogeneity in infection-related outcomes and implant survival. Changes in perioperative protocols and infection-prevention strategies such as novel antimicrobial coated implants may have influenced infection outcomes, although additional benefit is not established for every intervention [61]. Evolving surgical and antimicrobial management of established PJI, with comprehensive treatment protocols and antibiotics may also have affected whether and when prosthesis exchange or removal was required [48]. Where oncologic treatment prolongs survival after reconstruction, reduced competing mortality provides greater opportunity for late aseptic loosening or structural failure, without necessarily implying poorer implant performance [34]. Such survival gains cannot be assumed across all diagnoses or treatment periods [24]. Improvements in survival from cancer diagnosis do not necessarily translate into longer survival after reconstruction or altered cause-specific failure hazards. These potential temporal influences were not evaluated separately and may be intertwined with differences in case mix, implant selection, and follow-up.

Although individual-site descriptive outcomes are provided, distal femoral, proximal tibial, and combined reconstructions were grouped in the adjusted infection model, limiting finer site-specific inference. The first-event framework did not capture recurrent complications or transitions between failure modes, and assignment of a single primary Henderson failure may have simplified complex clinical sequences. The limited numbers of Type II, III, and V events restricted subtype-specific modelling. Sex-specific differences in risk-factor associations were not formally assessed and cannot be excluded. Several analyses were exploratory, no adjustment for multiple comparisons was applied, and complete-case analysis may have introduced bias. Incomplete documentation of estimated blood loss and immune status limited assessment of these potential confounders. The infection model was not externally validated. Sparse observations at higher previous-operation counts and evidence of nonlinearity limit interpretation of the per-operation hazard ratios as a precise dose–response relationship; the finding primarily supports prior local surgery as an associated risk factor. Finally, standardized functional scores and patient-reported outcome measures were not consistently available, preventing assessment of function, quality of life, and return to activity.

## 5. Conclusions

Modular megaprosthetic reconstruction demonstrated acceptable long-term durability in this heterogeneous real-world cohort. Nevertheless, postoperative complications remained common and continued to accumulate during follow-up. Periprosthetic infection was the dominant first recorded failure mode, followed by soft-tissue failure, whereas aseptic loosening occurred mainly during longer-term follow-up. Previous surgeries at the operative site, longer operative time, and diabetes mellitus were independently associated with infection and remained stable after clinically relevant adjustment. These findings support individualized preoperative risk assessment and optimization, together with early surveillance for infection and soft-tissue failure and continued long-term monitoring for mechanical complications. Prospective multicenter studies incorporating standardized functional and patient-reported outcomes are needed to validate these associations and refine risk-based follow-up strategies.

## Figures and Tables

**Table 1:** Baseline, oncologic, and surgical characteristics of the cohort overall and by grouped anatomical site.

Characteristic	Overall Cohort (n = 340)	Proximal Humerus (n = 62)	Proximal Femur (n = 165)	Around the Knee (n = 113)
Demographic and clinical characteristics				
Age, years	55.2 ± 19.7 (range 6–89)	56.5 ± 19.5	61.9 ± 13.0	44.8 ± 23.4
Male sex	186 (54.7%)	35 (56.5%)	91 (55.2%)	60 (53.1%)
BMI, kg/m^2^	27.8 ± 4.7	25.8 ± 3.4	30.3 ± 3.8	25.3 ± 4.6
Diabetes mellitus	71 (20.9%)	16 (25.8%)	44 (26.7%)	11 (9.7%)
Active smoking	166 (48.8%)	34 (54.8%)	96 (58.2%)	36 (31.9%)
Clinical indication				
Primary malignant bone tumor	175 (51.5%)	38 (61.3%)	59 (35.8%)	78 (69.0%)
Metastatic disease	99 (29.1%)	14 (22.6%)	68 (41.2%)	17 (15.0%)
Revision/trauma	66 (19.4%)	10 (16.1%)	38 (23.0%)	18 (15.9%)
Oncologic treatment				
Chemotherapy administered	178 (52.4%)	28 (45.2%)	79 (47.9%)	71 (62.8%)
Radiotherapy administered	67 (19.7%)	8 (12.9%)	47 (28.5%)	12 (10.6%)
Prosthesis and surgical variables				
MUTARS system	213 (62.6%)	31 (50.0%)	108 (65.5%)	74 (65.5%)
Stanmore system	127 (37.4%)	31 (50.0%)	57 (34.5%)	39 (34.5%)
Silver-coated prosthesis	155 (45.6%)	30 (48.4%)	63 (38.2%)	62 (54.9%)
Cemented fixation	211 (62.1%)	35 (56.5%)	107 (64.8%)	69 (61.1%)
Prosthesis length, cm	16 (12–18)	11.5 (10–12.5)	16 (14–18)	16 (15–20)
Specimen dimensions, cm^3^	120 (84–162)	62 (44.5–96)	120.5 (96–160)	136 (108–180)
Operative time, min	150 (130–180)	145 (125–160)	150 (130–180)	180 (150–230)
Hospital stay, days	8 [6–13.5]	5 [5–7.5]	8 [7–15]	9 [7–15]
Follow-up duration, years	5 [3–9]	5 [3–9]	6 [2–8]	5.5 [3.5–10]

Values are presented as n (%), mean ± SD, or median (IQR), as appropriate. Around the knee includes distal femur, proximal tibia, and combined distal femur + proximal tibia reconstructions. Percentages are calculated within column. Variable-specific missingness applies where relevant. Charlson Comorbidity Index was available for a limited subset (n = 85) and was not included in the main site-stratified table. MUTARS: Modular Universal Tumor and Revision System (implantcast GmbH, Buxtehude, Germany); Stanmore: Modular Endoprosthetic Tumour System (Stanmore Implants Worldwide Ltd., Elstree, UK).

**Table 2:** Patient and implant survival estimates overall and by surgical indication.

Group	1-Year (%)	3-Year (%)	5-Year (%)	10-Year (%)
Patient survival				
Overall cohort	92.0	79.7	71.4	61.3
Primary tumor	94.3	88.0	80.1	73.3
Metastatic disease	84.9	56.6	40.7	28.7
Revision/trauma	97.0	90.6	90.6	74.2
Implant survival				
Overall cohort	88.7	82.8	78.0	70.3
Primary tumor	85.9	78.3	72.2	64.7
Metastatic disease	94.8	88.6	84.2	84.2
Revision/trauma	87.3	85.7	83.8	72.7

Values are Kaplan–Meier survival probabilities. Log-rank *p*-values for comparisons across surgical indication groups were *p* < 0.001 for patient survival and *p* = 0.058 for implant survival.

**Table 3:** Complication burden and primary recorded Henderson failure modes overall and by grouped anatomical site.

Characteristic	Overall Cohort (n = 340)	Proximal Humerus (n = 62)	Proximal Femur (n = 165)	Around the Knee (n = 113)
Patients with ≥1 complication	133 (39.1%)	29 (46.8%)	50 (30.3%)	54 (47.8%)
Type I—Soft-tissue failure	40 (11.8%)	15 (24.2%)	20 (12.1%)	5 (4.4%)
Type II—Aseptic loosening	17 (5.0%)	3 (4.8%)	2 (1.2%)	12 (10.6%)
Type III—Structural failure	10 (2.9%)	0 (0.0%)	3 (1.8%)	7 (6.2%)
Type IV—Infection	49 (14.4%)	3 (4.8%)	23 (13.9%)	23 (20.4%)
Type V—Tumor progression	17 (5.0%)	8 (12.9%)	2 (1.2%)	7 (6.2%)

## Data Availability

The data supporting the findings of this study are available from the corresponding author upon reasonable request, subject to institutional and ethical restrictions. The data are not publicly available to protect patient privacy and confidentiality.

## Supplementary Materials

The following supporting information can be downloaded at https://www.mdpi.com/article/10.3390/cancers18203278/s1: Table S1: Descriptive outcomes and primary Henderson failure modes by individual anatomical reconstruction site; Table S2: Univariable Cox proportional hazards analyses for any postoperative complication and selected Henderson failure modes; Figure S1: Patient selection flow diagram; Figure S2: Kaplan–Meier estimates of complication-free survival.
