# Supplementary material for: Survival, Failure Modes, and Risk Factors After Limb Salvage Surgery with Modular Megaprosthetic Reconstruction
Source: Cancers (Basel). 2026 Oct 12;18(20):3278. doi: 10.3390/cancers18203278 (PMC13702188; doi:10.3390/cancers18203278)
Supplement: Supplementary file 1 [file cancers-18-03278-s001.zip › Supplementary_Table_S1_Anatomical_Site_Outcomes.pdf]

# Supplementary Materials

Survival, Failure Modes, and Risk Factors After Limb Salvage Surgery with Modular Megaprosthetic Reconstruction

## Table S1. Descriptive outcomes and primary Henderson failure modes by individual anatomical reconstruction site.

| Characteristic | Proximal humerus (n = 62) | Proximal femur (n = 165) | Distal femur (n = 88) | Proximal tibia (n = 20) | Combined distal femur/proximal tibia (n = 5) |
|---|---|---|---|---|---|
| Median follow-up, years | 5.0 | 6.0 | 6.0 | 4.5 | 4.0 |
| Deaths | 20 (32.3%) | 59 (35.8%) | 20 (22.7%) | 6 (30.0%) | 1 (20.0%) |
| Implant failures | 16 (25.8%) | 23 (13.9%) | 32 (36.4%) | 6 (30.0%) | 1 (20.0%) |
| Patients with ≥1 complication | 29 (46.8%) | 50 (30.3%) | 42 (47.7%) | 10 (50.0%) | 2 (40.0%) |
| Type I – Soft-tissue failure | 15 (24.2%) | 20 (12.1%) | 4 (4.5%) | 1 (5.0%) | 0 (0.0%) |
| Type II – Aseptic loosening | 3 (4.8%) | 2 (1.2%) | 12 (13.6%) | 0 (0.0%) | 0 (0.0%) |
| Type III – Structural failure | 0 (0.0%) | 3 (1.8%) | 5 (5.7%) | 1 (5.0%) | 1 (20.0%) |
| Type IV – Infection | 3 (4.8%) | 23 (13.9%) | 18 (20.5%) | 5 (25.0%) | 0 (0.0%) |
| Type V – Tumor progression | 8 (12.9%) | 2 (1.2%) | 3 (3.4%) | 3 (15.0%) | 1 (20.0%) |

Values are n (%) unless otherwise indicated. Percentages are calculated within each anatomical site using the denominator in the column heading. The five groups together comprise the full cohort (n = 340).

Implant failure was defined as revision requiring exchange or removal of the prosthesis.

Henderson categories represent the first primary recorded failure; each patient with a complication is counted once across Types I–V. Subsequent or recurrent events are not included.

Event percentages are crude observed proportions, not Kaplan–Meier survival or cumulative-incidence estimates. No formal between-site hypothesis testing was performed.
