# Supplementary material for: Survival, Failure Modes, and Risk Factors After Limb Salvage Surgery with Modular Megaprosthetic Reconstruction
Source: Cancers (Basel). 2026 Oct 12;18(20):3278. doi: 10.3390/cancers18203278 (PMC13702188; doi:10.3390/cancers18203278)
Supplement: Supplementary file 1 [file cancers-18-03278-s001.zip › Supplementary_Table_S2_Univariable_Cox_Analyses.pdf]

Supplementary Materials

Survival, Failure Modes, and Risk Factors After Limb Salvage Surgery with Modular Megaprosthetic Reconstruction

Table S2. Univariable Cox proportional hazards analyses for any postoperative complication and selected Henderson failure modes

Panel A. Any postoperative complication

Analysis population: overall cohort, n = 340; 133 events.

Variable	Hazard ratio	95% confidence interval	*p* value
Age, per year	1.0002	0.992–1.009	0.972
Prosthesis system: Stanmore vs MUTARS	0.75	0.52–1.09	0.125
Metastatic disease vs primary tumor	0.89	0.59–1.34	0.567
Revision/trauma vs primary tumor	0.82	0.52–1.30	0.403
Chemotherapy	1.07	0.76–1.51	0.688
Radiotherapy	1.29	0.85–1.98	0.236
Specimen dimensions, per cm^3	1.003	1.001–1.006	0.013
Prosthesis length, per cm	1.02	0.98–1.06	0.410
Hospital stay, per day	1.04	1.02–1.05	<0.001
Active smoking	1.27	0.90–1.79	0.172
Diabetes mellitus	1.84	1.25–2.69	0.002
BMI, per kg/m^2	1.02	0.98–1.06	0.307
Operative time, per minute	1.004	1.002–1.007	0.001

Hospital stay was measured postoperatively and should be interpreted as an associated marker of postoperative course and case complexity rather than as a baseline causal predictor.

Panel B. Type I soft-tissue failure

Restricted analysis population: proximal humeral and proximal femoral reconstructions, n = 227; 35 Type I events.

Variable	Hazard ratio	95% confidence interval	*p* value
Age, per year	1.019	0.995–1.044	0.125
Metastatic disease vs primary tumor	1.14	0.54–2.43	0.728
Revision/trauma vs primary tumor	1.47	0.49–2.80	0.716
Radiotherapy	1.47	0.70–3.06	0.306
Specimen dimensions, per cm^3	1.01	0.99–1.01	0.492
Anatomic vs reverse proximal humeral design	7.10	2.00–25.40	0.002
Dual-mobility vs hemiarthroplasty reconstruction	0.55	0.13–2.36	0.417

Design-specific variables applied only to the corresponding anatomical reconstruction. Primary tumor, reverse proximal humeral design, and hemiarthroplasty were the reference categories, as applicable.

Panel C. Type II aseptic loosening

Restricted analysis population: proximal humeral, distal femoral, and combined distal femoral/proximal tibial reconstructions, n = 152; 15 Type II events.

Variable	Hazard ratio	95% confidence interval	*p* value
Age, per year	0.98	0.96–1.01	0.233
Prosthesis system: Stanmore vs MUTARS	0.30	0.08–1.08	0.065
Chemotherapy	1.70	0.57–5.07	0.343
Prosthesis length, per cm	1.08	0.97–1.19	0.154

MUTARS was the reference prosthesis system. No predictor reached conventional statistical significance in this restricted analysis.

Panel D. Type IV infection

Analysis population: overall cohort; 49 Type IV infection events. Variable-specific denominators differed because complete-case analysis was performed separately for each univariable model.

Variable	Hazard ratio	95% confidence interval	*p* value
Age, per year	1.001	0.987–1.015	0.889
Prosthesis system: Stanmore vs MUTARS	0.67	0.36–1.24	0.204
Trevira use	1.29	0.72–2.31	0.400
Silver coating	0.74	0.41–1.33	0.311
Operative time, per minute	1.008	1.005–1.011	<0.001
Previous surgeries at the operative site, per additional surgery	2.63	2.20–3.14	<0.001
Cemented fixation	0.88	0.49–1.56	0.651
Active smoking	1.14	0.65–1.99	0.656
Diabetes mellitus	3.54	2.01–6.22	<0.001
BMI, per kg/m²	1.07	1.01–1.13	0.036
High vs low patient functional demand	1.04	0.59–1.84	0.896
Stem-to-bone length ratio	14.40	0.01–142,608	0.570
Stem-to-outer cortex width ratio	3.79	0.02–645	0.611
Charlson Comorbidity Index, per point	1.15	0.99–1.33	0.076
ASA score, per category	1.39	1.09–1.79	0.009
Transfusions, per additional unit	1.43	1.26–1.63	<0.001

MUTARS, no Trevira use, no silver coating, uncemented fixation, non-active smoking, absence of diabetes, and low functional demand were the reference categories, where applicable.

Charlson Comorbidity Index data were available for only 85 patients; this estimate should therefore be interpreted cautiously.

General notes

- Abbreviations: ASA, American Society of Anesthesiologists physical status; BMI, body mass index; CI, confidence interval; HR, hazard ratio.
- Hazard ratios for continuous variables are expressed per one-unit increase unless otherwise specified.
- All models were univariable Cox proportional hazards models. Subtype-specific analyses were exploratory, and no formal adjustment for multiple comparisons was performed.
- Type III structural failure was not subjected to risk-factor modelling because only 10 events occurred.
- Complete-case analysis was used, and denominators therefore varied across predictors with missing data.
